# Evaluation of nanodosimetric quantities for ion radiotherapy treatment planning based on the degree of association of survival with cluster dose

**DOI:** 10.1088/1361-6560/ae07a3

**Published:** 2025-09-25

**Authors:** Ramon Ortiz, José Ramos-Méndez, Jian-Hua Mao, Reinhard Schulte, Bruce Faddegon

**Affiliations:** 1 Department of Radiation Oncology, University of California San Francisco, San Francisco, CA, United States of America; 2 Lawrence Berkeley National Laboratory, Berkely, CA, United States of America; 3 Loma Linda University, School of Medicine, Loma Linda, CA, United States of America

**Keywords:** nanodosimetry, cluster dose, ICSD, particle therapy, cell survival

## Abstract

*Objective.* To demonstrate potential for a close association between cell survival and cluster dose for ionization parameters (*I*_p_), and to investigate the means to quantify the degree of this association when calculating cluster dose using these nanodosimetric quantities. *Approach.* The definitions of *I*_p_ considered were the number of clusters of *k* or more ionizations per unit track length {*C_k_, k*= 1,…10}. For this *I*_p_ definition, cluster dose is the number of clusters of *k* or more ionizations per unit mass. Three sets of published cell survival data, covering a range of clinically relevant particle types and energies, normal and tumor human cells, and aerobic and hypoxic conditions, were used to assess these *I*_p_. Values of *C_k_* were previously calculated for this survival data and evaluated for their application in treatment planning. New to this study, the dependence of cell survival on cluster dose, calculated as local fluence times the mean mass *I*_p_, was used. The degree of association of cell survival with cluster dose was quantified using three statistical methods: the moving window method, the residuals of linear quadratic fit, and the Bayesian information criteria. *Results.* all three methods identified *C*_5_ as the most closely associated with cell survival under aerobic conditions, and *C*_7_ under hypoxic conditions, consistent with visual observations. Remarkably, *C_k_* preferred for their close association with cell survival for different particle types having the same fluence, compared to alternative definitions, resulted in a statistically significant closer association of cell survival with cluster dose, regardless of particle fluence. *Significance.* fluence is a critical property Cluster dose has the potential of supplementing or even replacing RBE-weighted dose in optimization of ion therapy treatment plans. The proposed methodology lays the groundwork for rigorous identification of *I*_p_ that exhibit the highest degree of association of cell survival with cluster dose, a trait that greatly enhances the potential clinical impact of cluster dose.

## 1. Introduction

Nanodosimetric quantities are critical for the understanding the action of radiation to living tissues. Nanodosimetry studies the spatial distribution of energy deposition events produced by radiation (primarily ionization events) in biologically relevant nanoscopic volumes, such as volumes resembling short segments of DNA (Nettelbeck and Rabus 2011). There is a strong association between nanodosimetric quantities and various biological effects relevant to cancer therapy, including cell inactivation, DNA double-strand break cross-sections, and residual DNA damage (Nettelbeck and Rabus 2011, Conte *et al* 2012, 2018, Faddegon *et al* 2023, Mietelska *et al* 2024). These findings highlight the potential for using nanodosimetric quantities to enhance biologically optimized ion radiotherapy (Rucinski *et al* 2021). Multiple efforts have been made to integrate nanodosimetric considerations into radiotherapy treatment planning (RTP) strategies. These include incorporating nanodosimetric quantities into relative biological effectiveness (RBE) models (Villegas *et al* 2016, Dai *et al* 2020) and optimizing RBE-weighted absorbed dose and nanodosimetric quantities simultaneously (Ramos-Méndez *et al* 2018, Burigo *et al* 2019).

To provide a practical means to incorporate nanodosimetric quantities in RTP, an ionization detail (ID)-based mathematical formalism has been proposed (Faddegon *et al* 2023). This approach is based on the generalized definition of ionization cluster size *ν* and frequency distribution f(*ν*). An operator *G* collapses f(*ν*), usually the frequency ionization cluster size distribution fICSD(*ν*) for a particular particle type of a particular energy, into a scalar or vector quantity known as the ionization parameter *I*_p_. These quantities are defined in the formalism per particle and unit track length within the nanoscopic volume used for their calculation. To address the practicalities of current RTP strategies, such as optimizing the source fluence of individual pencil beams, an additional quantity is introduced: the generalized ionization cluster size dose (or cluster dose). Cluster dose is the product of the fluence of charged particles in a macroscopic volume (voxel) and the voxel-averaged *I*_p_. This quantity has already proven suitable for optimization by adjusting beam weights to deliver constant cluster dose distributions (Ortiz and Faddegon 2024). More detailed descriptions of these quantities can be found elsewhere (Faddegon *et al* 2023) and in section 2.1.

Previous efforts have discriminated between different definitions of *I*_p_ by comparing their degree of association with cell survival at constant fluence (Faddegon *et al* 2023). Preferred *I*_p_ are those with *I*_p_ definitions that, for particles with the same fluence, result in a close association of biological effect with the biological endpoint under study across different particle types and energies. That is, for preferred *I*_p_, particles with the same values of *I**_p_*** and fluence are comparable in beam quality, leading to nearly identical biological effects. One definition of *I*_p_ considered in the study was *C_k_*, the number of nanoscopic ionization clusters of *k* or more ionizations per unit track length. Those efforts identified that the preferred *C_k_* for the cell survival data considered were *C*_5_ and *C*_7_ in aerobic and hypoxic conditions, respectively.

The present work extends the theoretical framework of the ID-based formalism, presented in (Faddegon *et al* 2023), by introducing cell survival curves vs. cluster dose and demonstrating, for the first time, a remarkably close association between cluster dose, derived from the preferred I_p_, and cell survival, independent of the particle fluence and *I*_p_ value. This is a key advance because, unlike most nanodosimetric quantities, cluster dose is inherently optimizable, analogous to absorbed dose, and therefore suitable for integration into RTP. This is an important property of preferred *I*_p_.

In this study, we establish a methodology to identify a preferred I_p_ definitions that ensures a ‘same cluster dose, same survival’ relationship, independent of particle type and energy. First, we introduce the approach of plotting biological effect against cluster dose calculated with different *I*_p_, using published cell survival data for normal and tumor cells irradiated under aerobic and hypoxic conditions with a wide range of particle types and energies. We then evaluate the ‘tightness’ of these plots for {*C_k_, k* = 1,…10} using different statistical approaches.

## 2. Materials and methods

### 2.1. Ionization detail parameter and cluster dose

This section provides a concise introduction to the nanodosimetric concepts and derived quantities relevant to this work. A detailed description of the ID formalism and the methods for calculating ID quantities can be found in our previous work (Faddegon *et al* 2023).

In this work, we consider a practical approach to plan proton and ion radiation treatments based solely on nanodosimetry (Faddegon *et al* 2023). Unlike RBE-based models, which require specific weighting factors that often lead to varying predictions of isoeffective dose with no established consensus, the nanodosimetric approach to RTP using cluster dose does not require an RBE-like weighting factor.

Ionization clusters are the set of ionizations created in nanoscopic volumes resembling short segments of DNA where complex damage may occur. Since energy deposition by radiation in matter is a stochastic process, the size of ionization clusters is described by the fICSD $f\left( \nu \right)$, where $\nu $ is the cluster size. The fICSDs are computed using TOPAS-nBio (Schuemann *et al* 2018) to simulate a 161 nm long × 30.4 nm rectangular monoenergetic parallel beam of source particles incident on the geometry as described elsewhere (Faddegon *et al* 2023). In short, this geometry consists of 1800 small cylinders (sampling volumes or targets) placed with random position and orientation without overlap within a larger cylinder (sensitive volume) of radius 30.4 nm and length 161 nm, representing a short segment of chromatin. The fICSD for a given particle type and energy fICSD is computed by adding up the number of clusters of size ν from all (monoenergetic) source particles over these 1800 sensitive volumes and dividing by the number of source particles and the average track length of the particles (calculated as the mean cord length of the larger cylinder). The small cylinders are of radius 2.3 nm and length 3.4 nm, representing 10 base-pair-long DNA segments. A single particle traversing the larger cylinder produces varying numbers of ionizations in the individual small cylinders. Here, ν is the cluster size (number of ionizations) in a single small cylinder. The frequency distribution of these cluster sizes for a given particle type and energy fICSD is computed by adding up the number of clusters of size ν from all (monoenergetic) source particles and dividing by the number of source particles and the average track length of the particles (calculated as the mean cord length of the larger cylinder).

To collapse the characteristics of these frequency distributions into a single scalar quantity, the ${I_{\text{p}}}$ is derived from $f\left( \nu \right)$ by applying a specific operator. An example of ${I_{\text{p}}}$, and the one considered in this work due to its potential association with cell survival found previously (Faddegon *et al* 2023), is the number of clusters of *k* or more ionizations:

\begin{align*}{{\text{C}}_{\text{k}}} = \mathop \sum \limits_{{{\nu = k}}}^{{{{\nu }}_{{\text{max}}}}} {{ f\left(\nu \right)}}\,.\end{align*}

Note that *C_k_* was denoted as *F_k_* in previous publications (Faddegon *et al* 2023, Ortiz and Faddegon 2024). The quantities $f\left( \nu \right)$ and ${I_{\text{p}}}$ are calculated for each particle class (i.e. type and energy) per track length through the nanoscopic scoring volume, and per particle, making them independent of particle fluence. The nanodosimetric quantities fICSD and *I*_p_ are normalized by the track length to facilitate their use in treatment planning. Then, the voxel-averaged *I*_p_, a macroscopic quantity used in treatment planning, is conveniently proportional to cluster dose as follows.

To address the practicalities of RTP, specifically, the use of macroscopic millimeter-sized volumes (voxels) and optimization of the particle beam fluence to shape the distributions of a given quantity in patient volumes, two quantities were defined: the voxel-averaged ${I_{\text{p}}}$ and cluster dose. The voxel-averaged ${I_{\text{p}}}$ is calculated in millimeter-size voxels using condensed-history Monte Carlo simulations. In these macroscopic voxels, a particle may lose enough energy to have more than one value of ${I_{\text{p}}}$ (or fICSD) in the same voxel. Charged particles can also be generated or stop in the voxel. To account for this, the track of each particle in a voxel is subdivided into shorter track segments, with each segment corresponding to a single particle class *c*, and the track length of the particles in each class summed up to yield $t_j^c$. The voxel-averaged ${I_{\text{p}}}$ (or fICSD) is then calculated using equation (2):

\begin{align*}{\text{I}}_{\text{p}}^{{{{\varphi }}_{\text{j}}}} = \frac{{\mathop \sum \nolimits_{{\text{c}}\epsilon {{{\varphi }}_{\text{j}}}} {\text{t}}_{\text{j}}^{\text{c}}{\text{I}}_{\text{p}}^{\text{c}}}}{{\mathop \sum \nolimits_{{\text{c}}\epsilon {{{\varphi }}_{\text{j}}}} {\text{t}}_{\text{j}}^{\text{c}}}}{\text{,}}\end{align*}

where $\varphi $ is the set of charged particles interacting in the voxel *j*. This calculation includes contributions from both primary ions and secondary particles, including fragments generated via nuclear fragmentation. Although secondary fragments with energies beyond the database limits can be produced, additional simulations confirmed that they constitute less than 1% of the total fluence contributing to *I*_p_. For those particles, the *I*_p_ associated with the nearest boundary value (i.e. the lowest or highest available energy) was used.

From this macroscopic ${I_{\text{p}}}$, the cluster dose, $g_j^{\left( {{I_{\text{p}}}} \right)}$, in each voxel is defined as the product of the fluence of charged particles, ${\phi _j}$, and the mean mass ${I_{\text{p}}}$, i.e. $I_{\text{p}}^{{{{\varphi }}_j}}$ divided by the density of the material used to compute the nanoscopic $f\left( \nu \right)$, ${\rho _0}$, as:

\begin{align*}{\text{g}}_{\text{j}}^{({{\text{I}}_{\text{p}}})}: = {\phi _{\text{j}}}{\text{ I}}_{\text{p}}^{{{{\varphi }}_{\text{j}}}}/{{{\rho }}_{\text{0}}}{\text{}}.\end{align*}

This approach enables us to compute the total number of ionization clusters produced by the set of particles interacting within a patient treated with clinical proton or ion beams; that is, cluster dose distributions. These can be optimized for treatment planning using the same codes used for conventional treatment planning.

Fluence was computed as the total track length of particles interacting within a voxel divided by the voxel’s volume, including primary and secondary charged particles contributing to the ${I_p}$.

### 2.2. Cluster dose survival curves

Cluster dose survival curves were determined from published measured *in vitro* cell survival curves plotted against absorbed dose and cluster dose, the latter values computed from the absorbed dose using Monte Carlo simulations. Three sets of *in vitro* cell survival data were chosen to cover a wide range of particle types and energies from both research and clinical beams. Measurements were done at two different institutions by different investigators under aerobic and hypoxic conditions for a tumor and a non-tumor cell line. Our close, continued collaboration with the scientists who measured the data greatly facilitated our ability to reproduce experimental beam conditions to calculate *I*_p_ and cluster dose accurately at the position of the cell samples. The first and second sets involve the survival of human kidney T-1 cells measured at the Lawrence Berkeley National Laboratory at various points along the pristine passively scattered Bragg curve of carbon, neon and argon beams with initial energies of 400, 425 and 570 MeV u^−1^, respectively, in aerobic and hypoxic oxygenation conditions (Blakely *et al* 1979). These two datasets will be referred hereafter as *Blakely1979Aerobic* and *Blakely1979Hypoxic*. The third set involves survival of human alveolar adenocarcinoma A549 cells measured at the Heidelberg Ion Beam Therapy Center at the center of a scanned 1 cm spread out Bragg peak (SOBP) of clinical proton, helium, carbon and oxygen beams (Dokic *et al* 2016). This dataset will be referred hereafter as *Dokic2016Aerobic*.

**Figure 1.**
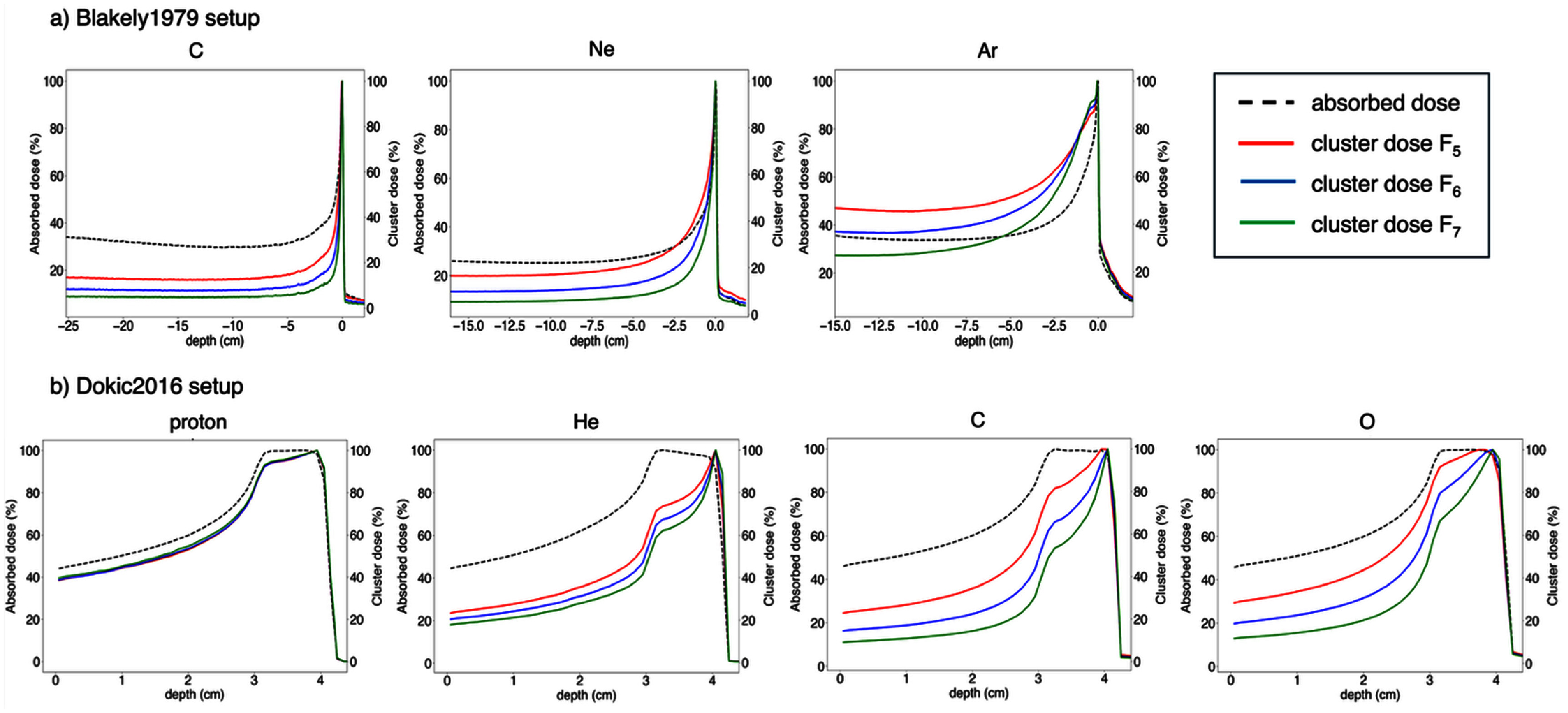
Depth distributions of absorbed dose and cluster dose, computed from *C*_5_*, C*_6_*, C*_7_, corresponding to the *Blakely*1979 and *Dokic*2016 experiments.

Cluster dose was calculated using the TOPAS Monte Carlo toolkit (Perl *et al* 2012, Faddegon *et al* 2020). The version used in this work was OpenTOPAS v.4.0, available on the TOPAS collaboration GitHub (https://github.com/OpenTOPAS). Standard Geant4 physics cross-section data files were used from the physics list built using the Geant4_Modular option with ‘g4em-standard_opt4’ ‘g4h-phy_QGSP_BIC_HP’ ‘g4decay’ ‘g4ion-binarycascade’ ‘g4h-elastic_HP’ ‘g4stopping’ modules. All transportation parameters used default options (Geant4 Collaboration 2021). The experimental setups described above were reproduced in the simulations. Absorbed dose and cluster dose depth distributions for each setup are shown in figure 1. The cluster dose at each experimental measurement point (depth) was calculated as described in section 2.1 and in previous works (Faddegon *et al* 2023, Ortiz and Faddegon 2024). The scoring resolution in depth was 1 mm. The particle fluence at each experimental dose point, used to compute the cluster dose, was derived from the dose per fluence value obtained from simulations and the reported dose received by the cells at each measurement point. The maximum energies of the different ions employed in these datasets are within the highest energy limits of the fICSD database used in this study, as described in detail in (Faddegon *et al* 2023).

Survival (in logarithmic scale) at each measurement point, dose level, and particle type were plotted against the corresponding cluster dose values.

Cluster dose survival curves were calculated for cluster dose definitions derived from *C_k_* parameters with *k* from 1 to 10 (see section 2.1). The rationale for considering *C_k_* in this work is that previous studies have shown that this definition of *I*_p_ closely associates with cell survival (Faddegon *et al* 2023).

### 2.3. Quantification of the degree of association of cell survival with cluster dose

In this work, we use three different statistical approaches to quantify the degree of association of survival with cluster dose based on minimizing the dispersion of survival values for a given cluster dose value, independent of particle type, energy and fluence. These approaches are applicable to other biological endpoints as well. Method 1 may be applied to biological endpoints where the association with cluster dose does not follow a known response curve, Method 2 to endpoints where the shape of the response curve is known. Method 3 is useful for comparing different response models.

Two of these statistical approaches rely on a linear-quadratic (LQ) dependence of the survival on cluster dose. For these approaches, an LQ curve with *ɑ* and *β* parameters was fit to the cluster dose survival data:

\begin{align*}{\text{lnS}} = - \alpha {\text{g}} - \beta {{\text{g}}^{\text{2}}}{\text{,}}\end{align*}

where *S* is the cell survival and *g* is the cluster dose. A weighting factor of the reciprocal of the survival (1/survival) was applied in the LQ fitting procedure.

The rationale for the survival curve for a given beam quality (particle type and energy) to follow an LQ dependence with cluster dose, as it does with absorbed dose (McMahon 2018), is as follows. In a charged particle beam, absorbed dose equals the product of particle fluence and the mean restricted mass collisional stopping power. Similarly, cluster dose equals the product of particle fluence and the mean mass *I*_p_. In a specific irradiated region, such as a given depth in a particle SOBP, the stopping power is independent of dose and the value of the *I*_p_ is independent of cluster dose. Thus, the survival in both cases depends only on fluence, and the survival curve will have the same shape whether plotted against absorbed dose or cluster dose. Furthermore, the LQ parameters for the survival curve as a function of absorbed dose depends on particle type and LET. A key property of preferred *I*_p_ is, given the same fluence, the survival for a given *I*_p_ is independent of particle type and LET. For treatment planning purposes, we seek preferred *I*_p_ for which the survival for a given cluster dose is also independent of fluence. In this ideal case, results for different particle types and energy for a given cell type and environment will have the same LQ parameters. The further the selected *I*_p_ varies from this ideal, the greater the individual points on the survival versus cluster dose curve deviate from the LQ fit to the data. In this work, we exploit this method to evaluate preferred *I*_p_ for their use in treatment planning.

#### 2.3.1. Method 1: moving window method

The *moving window* (also called *moving average*) method (Keller and Massart 1991) was implemented as follows. The survival data were first ordered based on increasing cluster dose values to compare survival dispersion across cluster dose ranges. Then, the standard deviation of survival values was calculated over limited ranges of cluster dose values, denoted as *windows*. In this study, the window size (the number of survival points in the window) was set to 1% of the total number of survival points in the dataset, with a minimum of three datapoints. The use of different window sizes was also explored. The windowing process begins with the survival data point at the lowest cluster dose. The window was then iteratively shifted by removing the lowest cluster data point and adding the next highest cluster dose data point beyond the current window. This process continued until the highest cluster dose in the dataset was included in a window. Each window centered on a datapoint and maintained symmetry, containing equal number of preceding and following datapoints. This method enables a quasi-continuous assessment of survival dispersion throughout the dataset.

The standard deviation in each window (${\sigma _w}$) was calculated using equation (5),

\begin{align*}{\sigma _{\text{w}}} = \sqrt {\frac{{\text{1}}}{n}{{\sum\nolimits_{i{{ = 1}}}^n {\left( {{S_i} - \overline {{\text{ }}S} } \right)} }^2}} ,\end{align*}

where *n* is the number of datapoints in the window, *S_i_* are the survival datapoints, and $\bar S$ the mean of the survival data in the window. Since the window size was constant, the average standard deviation in the entire dataset (${\sigma _T}$) was calculated as the mean of the standard deviation of each window (${\sigma _w}$) using equation (6),

\begin{align*}{{{\sigma }}_{\text{T}}} = \frac{{\mathop \sum \nolimits_{{\text{w}} = 1}^{\text{N}} {{{\sigma }}_{\text{w}}}{ }}}{{\text{N}}}{\text{,}}\end{align*}

where *N* is the number of windows. Finally, the standard error of the average standard deviation (${\text{S}}{{\text{E}}_T}$), was calculated as:

\begin{align*}{\text{S}}{{\text{E}}_T} = \frac{{\sqrt {\frac{{\text{1}}}{N}{{\mathop \sum \nolimits}}_{w{{ = 1}}}^N{{\left( {{\sigma _w} - \overline {{\sigma _w}} } \right)}^{\text{2}}}} }}{{\sqrt N }}{\text{,}}\end{align*}

where the numerator is the standard deviation of ${\sigma _T}$, and *N* the number of windows. One standard deviation was considered.

The average standard deviation in the entire dataset (${\sigma _T}$), referred hereafter as the dispersion of survival values, was used to identify the preferred *I*_p_. The *I*_p_ for which the dispersion of survival values in cluster dose survival curves was the lowest was considered the preferred *I*_p_.

#### 2.3.2. Method 2: residuals from LQ model fit

An LQ curve was fit to the cluster dose survival data for each *I*_p_ considered. The LQ fit was performed using the SciPy Python library (Virtanen *et al* 2020). The relative residuals of the survival points to the fitted curve were computed. In other words, the absolute difference of each point to the survival predicted by the LQ fit for each cluster dose point was evaluated and divided by the survival value. The average residual in the entire dataset (${r_T}$) was computed as the mean of the residual of each datapoint (${r_i}$):

\begin{align*}{r_T} = \,\frac{{\mathop \sum \nolimits_{i = 1}^N {r_i}}}{N} = \frac{{\mathop \sum \nolimits_{i = 1}^N \left(|{S_i} - {S_{{i_\text{fit}}}}|\right)/{S_{{i_\text{fit}}}}\,}}{N},\end{align*}

where *S_i_* are the survival datapoints, ${S_{{i_{fit}}}}$ is the survival datapoints derived from the LQ fit, and *N* is the number of datapoints in the dataset. Finally, the standard error of the average residual (${\text{S}}{{\text{E}}_T}$), was calculated as:

\begin{align*}{\text{S}}{{\text{E}}_T}{\text{ = }}\frac{{\sqrt {\frac{{\text{1}}}{N}{{\mathop \sum \nolimits}}_{i{\text{ = 1}}}^N{{\left( {{r_i} - \overline {{r_i}} } \right)}^{\text{2}}}} }}{{\sqrt N }}{\text{,}}\end{align*}

where the numerator is the standard deviation of ${r_T}$, and *N* the number of datapoints. One standard deviation was considered.

The *I*_p_ with the lowest average residual (${r_T}$), referred hereafter as the dispersion of survival values, was considered the preferred *I_p_*.

#### 2.3.3. Method 3: Bayesian information criterion (BIC) from LQ model fit

A linear mixed regression model of the outcome, ln(S) (see equation (2)), was used to assess its association with the cluster dose derived from each *I*_p_ in separate models. BIC (Bishop 1996) was used to identify the preferred *I*_p_ by finding the lowest BIC. BIC evaluates the likelihood of a given model to fit measured data with a penalty term for model complexity (i.e. number of model parameters), as shown in equation (10),

\begin{align*}{\text{BIC = LLH}} + {\text{k log}}\left( {\text{N}} \right){\text{,}}\end{align*}

where *N* is the number of datapoints, k the number of model parameters, and LLH is the −2-log likelihood, computed as shown in equation (11), assuming the normal distribution of residuals (Bishop 2006):

\begin{align*}{\text{LLH}}= - {\text{N log}}\left( {{\sigma }} \right){{ + }}\frac{{{{\text{r}}^{\text{2}}}}}{{{{{\sigma }}^{\text{2}}}}}{\text{,}}\end{align*}

where $r$ is the sum of squared residuals from the LQ fit (see equation (8)), and $\sigma $ the standard deviation of residuals.

To estimate the standard error of the BIC, we adopted the bootstrapping approach (Riffenburgh and Gillen 2020) as follows. From each dataset, we generated 100 samples containing 80% of datapoints randomly selected from the original dataset. For each sample, the BIC was calculated (${\text{BI}}{{\text{C}}_i}$). The average BIC of the entire dataset was computed as the mean of the BIC of each sample, and the standard error of the average BIC was computed as shown in equation (12):

\begin{align*}{\text{S}}{{\text{E}}_{\text{T}}} = \frac{{\sqrt {\frac{{\text{1}}}{{\text{N}}}\mathop \sum \nolimits_{{\text{i}} = 1}^{\text{N}} {{\left( {{\text{BI}}{{\text{C}}_{\text{i}}}{{ - }}\overline {{\text{BI}}{{\text{C}}_{\text{i}}}} } \right)}^{\text{2}}}} }}{{\sqrt {\text{N}} }}\,,\end{align*}

where *N* is the number of samples (i.e. 100). One standard deviation was considered.

Using the BIC, we also compared a linear fit with the LQ fit and found that the LQ fit showed a lower BIC, and therefore, presented the results from that model. Separate models were generated for hypoxic and aerobic data, and thus a different *I*_p_ was selected for each.

## 3. Results

### 3.1. Cluster dose survival curves

**Figure 2.**
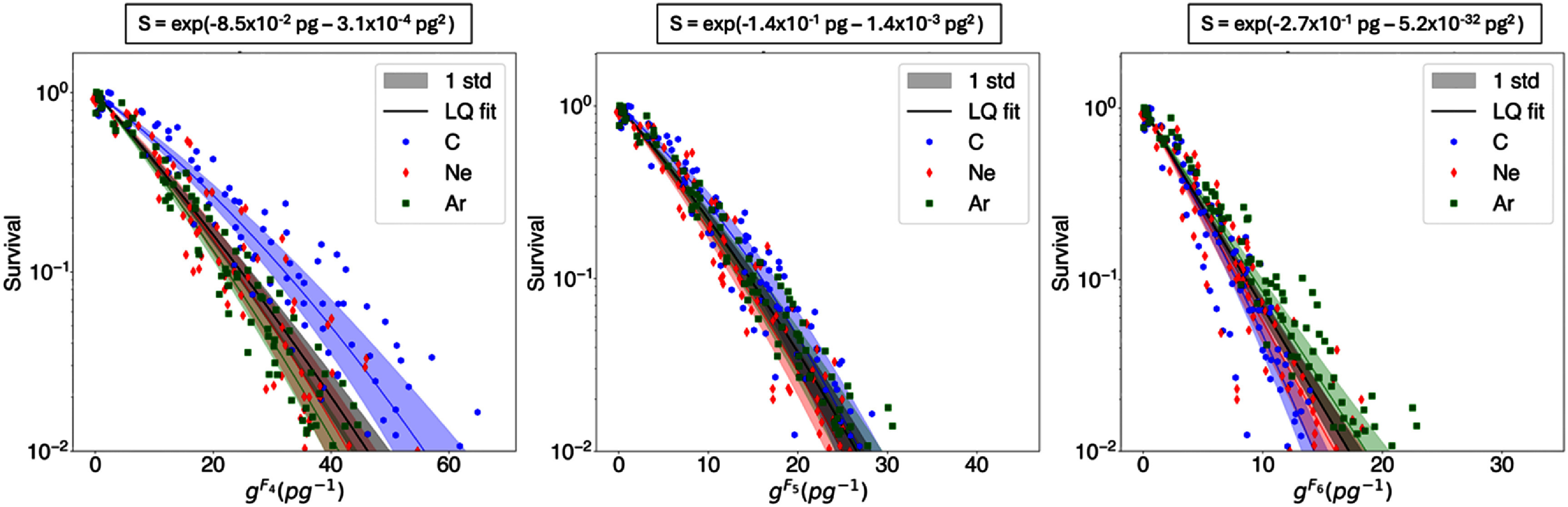
Survival vs. cluster dose (*g^Ck^*) computed from *C*_4_*, C*_5_, and *C*_6_ for dataset *Blakely*1979*Aerobic*. The solid lines are LQ fits for each ion (see legend colors) and for the entire dataset (in black). The color bands indicate the uncertainty associated with the fit (one standard deviation). The LQ fit equation for the entire dataset is shown at the top of each figure.

**Figure 3.**
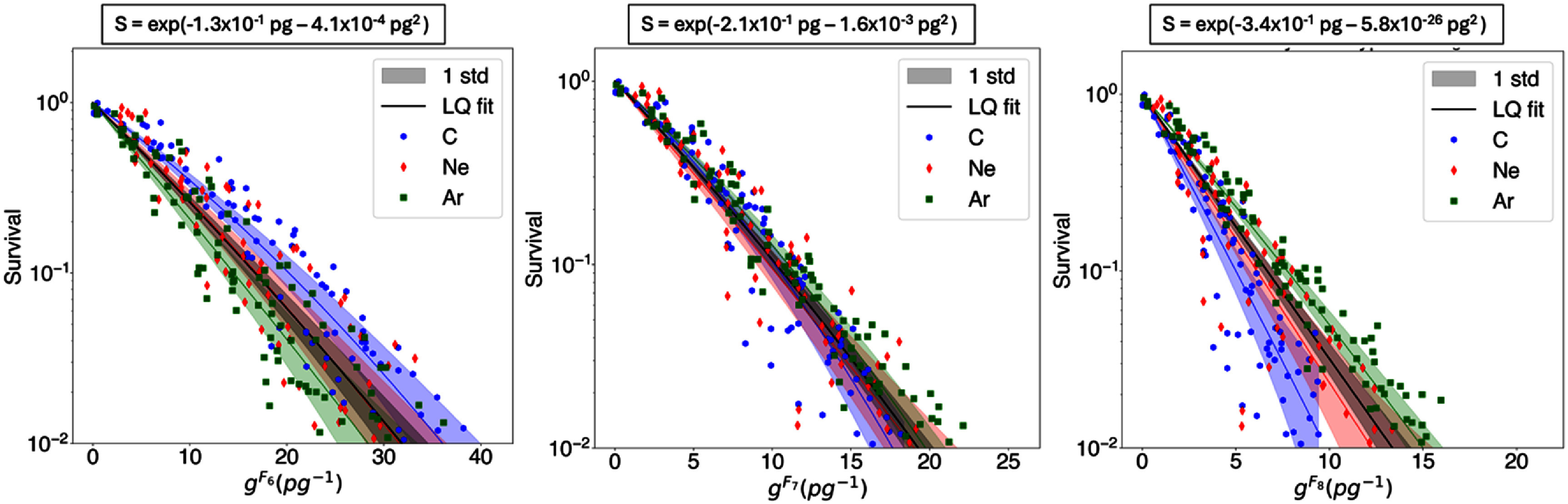
Survival vs. cluster dose (*g^Ck^*) computed from *C*_6_*, C*_7_, and *C*_8_ for dataset *Blakely*1979*Hypoxic*. The solid lines are LQ fits for each ion (see legend colors) and for the entire dataset (in black). The color bands indicate the uncertainty associated with the fit (one standard deviation). The LQ fit equation for the entire dataset is shown at the top of each figure.

**Figure 4.**
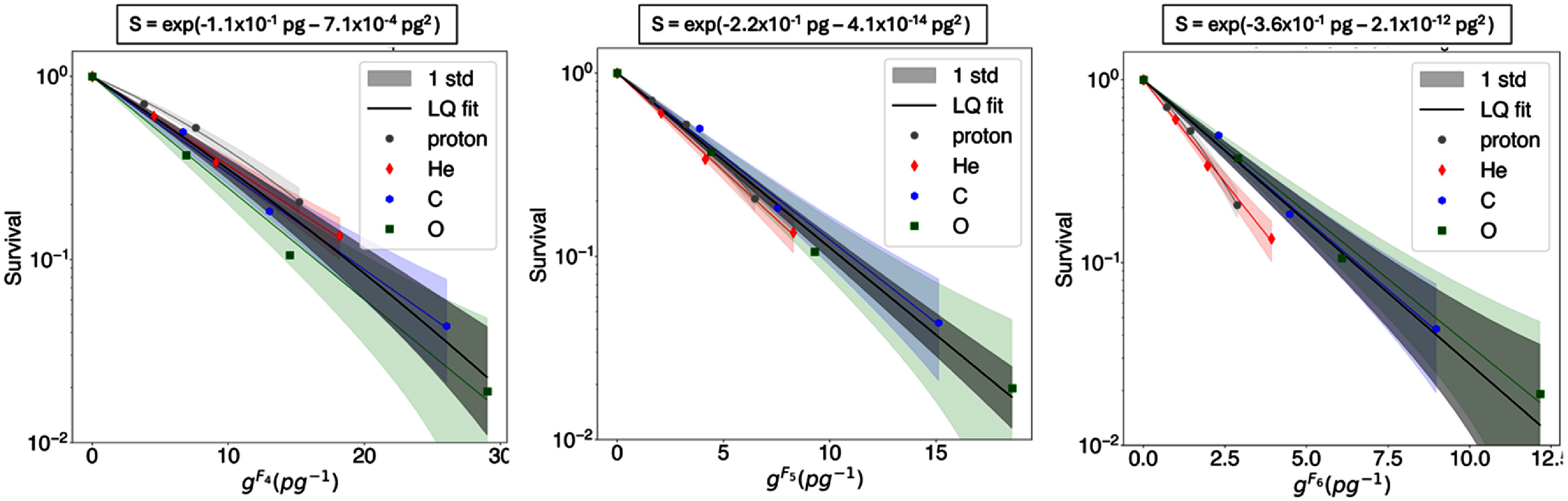
Survival vs. cluster dose (g^Ck^) computed from *C*_4_*, C*_5_, and *C*_6_ for dataset Dokic2016*Aerobic*. The solid lines are LQ fits for each ion (see legend colors) and for the entire dataset (in black). The color bands indicate the uncertainty associated with the fit (one standard deviation). The LQ fit equation for the entire dataset is shown at the top of each figure.

Cluster dose survival curves for the three datasets are shown in figures 2–4. The cluster dose units are pg^−1^, corresponding to the number of clusters of size *k* or more in a water cube of 1 *μ*m width. Results displayed are limited to the three *I*_p_ with consecutive cluster size *k* that exhibit the lowest survival dispersion based on visual inspection. As illustrated in figures 2–4, survival follows an LQ dependency with cluster dose (see equation (4)). The LQ fits for each particle type and for the entire dataset, along with the *α* and *β* parameters, are also presented in these figures.

**Figure 5.**
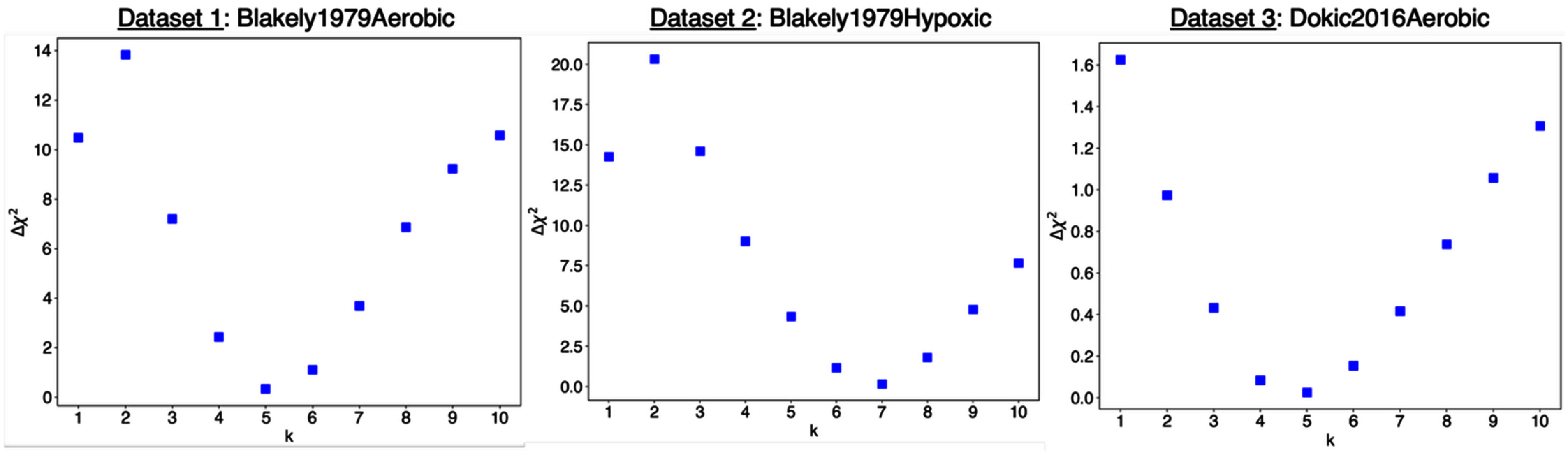
${{\Delta }}{{{\chi }}^{\text{2}}}$ values for various *C_k_* (with *k* from 1 to 10) and the three datasets studied.

By visual inspection, *C*_5_ seems to be the preferred *I*_p_ for aerobic datasets, and *C*_7_ for the hypoxic dataset. These plots show that for the preferred *I*_p_ the individual LQ fits for each particle overlap with the fit for the entire dataset within one standard deviation, whereas the LQ fits with other *I*_p_ definitions are outside of one standard deviation of the fit to the entire dataset. Standard deviation bands were estimated using random sampling, generating a thousand random *α* and *β* values based on their fitting errors, and deriving standard deviation bands from the resulting curves. Remarkably, for the cluster dose survival curves calculated considering these preferred *I*_p_, the variation in survival values for the same cluster dose is the lowest. Additionally, we assessed the statistical significance of this result by applying the chi-squared test to the LQ model fits. Specifically, we evaluated the difference of chi-squared values of individual and combined LQ fits:

\begin{align*}\Delta {\chi ^{\text{2}}} = \chi _{{\text{combined }}}^{\text{2}} - \chi _{{\text{individuals}}}^{\text{2}},\end{align*}

where ${{\chi }}_{{\text{individuals}}}^{\text{2}}$ is the sum of chi-squared values from independent fits to each dataset, and ${{\chi }}_{{\text{combined}}}^{\text{2}}$ the chi-squared value of the fit to the entire dataset. As shown in figure 5, for aerobic datasets, *C_5_* showed the lowest ${{\Delta }}{{{\chi }}^{\text{2}}}$ among the different *C_k_* tested, i.e. 0.33 for Blakely1979Aerobic, and 0.03 for Dokic2016Aerobic. For the hypoxic dataset (Blakely1979Hypoxic), *C*_7_ showed a minimal ${{\Delta }}{{{\chi }}^{\text{2}}}$ of 0.16.

### 3.2. Degree of association of survival with cluster dose

**Figure 6.**
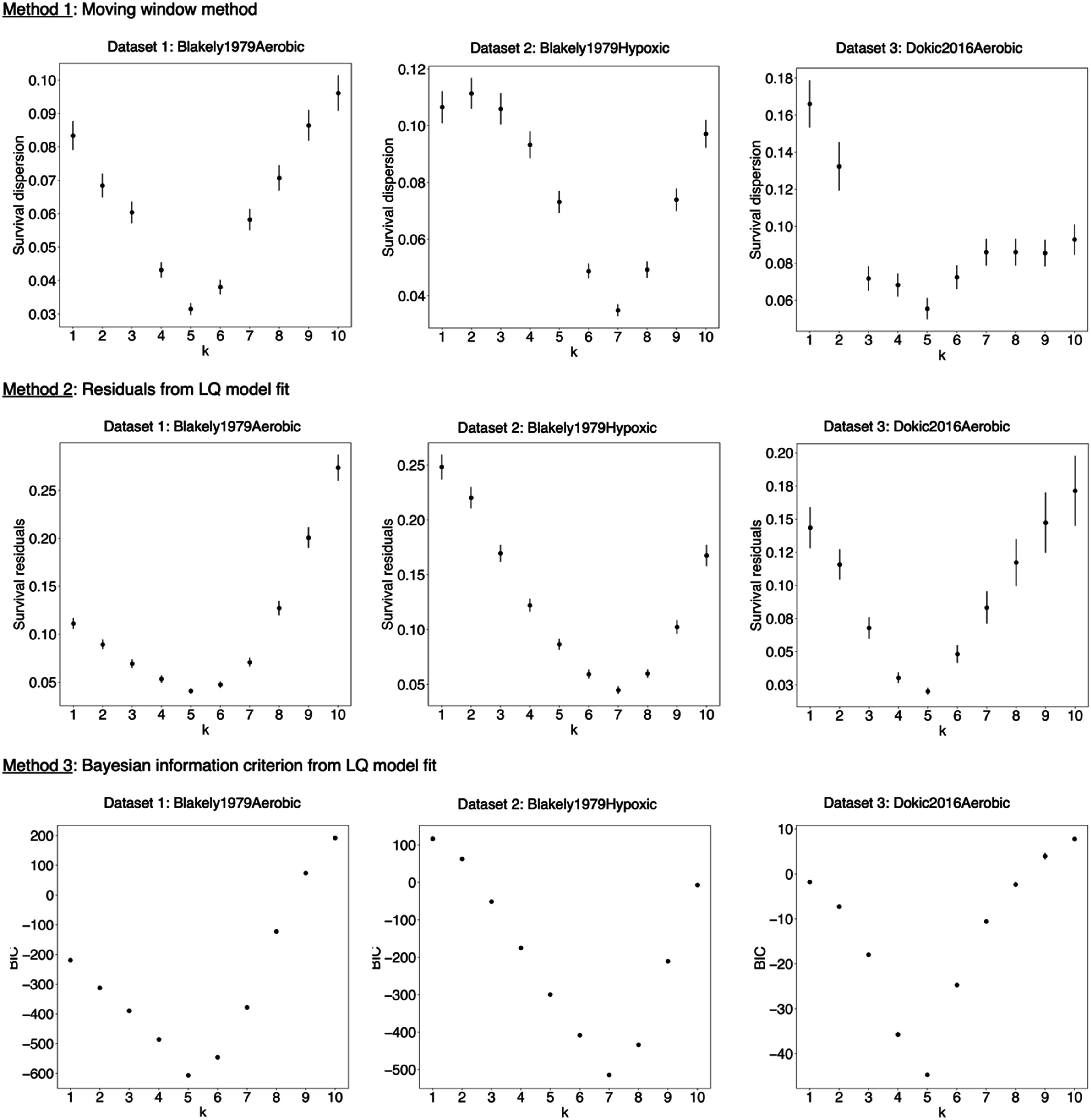
Dispersion of survival data for various *C_k_* (with *k* from 1 to 10) computed with Methods 1, 2, and 3. The error bars correspond to the standard error computed from one standard deviation.

The degree of association of cell survival with cluster dose was determined in terms of the dispersion of survival values for a given cluster dose value. This dispersion is defined as the average standard deviation in the entire dataset in Methods 1, the average residuals from the LQ fit in Method 2, and the BIC in Method 3. Figure 6 presents the dispersion values computed with these three methods for the set of *I*_p_ evaluated, i.e. {*C, k* = 1,…10} (see equation (2)).

The three statistical methods agree that the *I*_p_ that results in the lowest dispersion in survival values when survival is plotted against cluster dose, is *C*_5_ for aerobic datasets and *C*_7_ for the hypoxic dataset. That is, the survival dispersion in the cluster dose survival curves for these *I*_p_ is the smallest, considering its standard error (derived from one standard deviation). The finding that the preferred *I*_p_ from our previous study results in the least dispersion is statistically significant.

## 4. Discussion

Nanodosimetric approaches, including the ID approach, have already demonstrated their potential to advance the practices of ion RTP. A key clinical objective in nanodosimetry is to find a practical approach for RTP that accounts for biological effects pertaining to treatment outcome that result from the detailed spatial distribution of ionizations along charged particle paths; this is ID-based RTP (Faddegon *et al* 2023). Cluster dose is the product of charged particle fluence and mass *I*_p_. Similarly, absorbed dose in the product of charged particle fluence and restricted mass collision stopping power. Conventional planning is based on RBE-weighted absorbed dose. A new approach to planning has been proposed where cluster dose is used along with or even in place of RBE-weighted dose (section 4.5 of Faddegon *et al* (2023)). The scaling of cluster dose with fluence is a critical property for RTP, as it is for RBE-weighted dose. Then, cluster dose may be optimized by adjusting the weights of individual pencil beam spots (Ortiz and Faddegon 2024), as is typically done in current clinical treatment planning with RBE-weighted dose. We have shown that, with *I*_p_ defined in such a way as to result in a close association with cell survival, given the same particle fluence, cluster dose calculated with these *I*_p_ is also closely associates with cell survival, independent of particle fluence (figures 2–4). ID-based RTP with cluster dose, using these *I*_p_, can be done without consideration of RBE. This approach may possibly result in a comparable or perhaps better treatment plan than conventionally planned with RBE-weighted absorbed dose. However, we first need to select the best of the preferred *I*_p_ for use in treatment planning.

As a first approach, previous efforts evaluated preferred *I*_p_ that better associate with cell survival for particles of different type and energy, but the same fluence (Faddegon *et al* 2023). One might achieve practical treatment plans by constraining voxel-averaged preferred *I*_p_ to be constant across the target volume and minimized in organs at risk (see, e.g. (Burigo *et al* 2019)). However, requiring uniform voxel-averaged *I*_p_ limits the permitted mix of particle types and energies in a voxel and requires uniform charged particle fluence, which is difficult to achieve. Instead, should *I*_p_ exist that result in (close to) the same biological effect when delivering the same cluster dose, optimal ion therapy plans may be achieved solely by adopting constraints on cluster dose (e.g. Ortiz and Faddegon 2024). This constitutes a practical approach to RTP with nanodosimetric quantities alone, without the use of RBE.

The close association observed between cluster dose and cell survival in this study, albeit for a limited set of data, opens the door to an additional approach to evaluate *I*_p_ for ID-based RTP. In this work we proposed to distinguish between different definition of *I*_p_ based on the degree of association of cell survival with cluster dose calculated with these *I*_p_. Then, we proposed several methods to do so and demonstrated the use of these methods to further evaluate preferred *I*_p_ for their application in practical ID-based RTP.

We showed that the dependence of survival on cluster dose follows an LQ response model, as it does for the absorbed dose. This suggests the study of biological response to cluster dose may adopt concepts and models from the study of biological response to absorbed dose. For some datasets and *I*_p_ definitions, the relationship of survival with cluster dose was predominantly linear and the beta component could be neglected. We applied the LQ model throughout rather than assuming a linear response to avoid bias in the *I*_p_ evaluation. The use of the LQ model in this study stems from the fact that both absorbed dose and cluster dose scale with fluence for a specified radiation quality. This fluence dependence supports the use of a LQ survival model. Importantly, the proportionality factors differ. Absorbed dose scales with fluence via stopping power, while cluster dose scales with fluence through a selected *I*_p_, reflecting the stochastic nature of radiation interactions at the nanometric level. Consequently, while absorbed dose alone does not uniquely determine cell survival across different radiation qualities, cluster dose allows cell survival to be modeled more consistently, provided the *I*_p_ is appropriately selected. This approach effectively integrates radiation quality into the definition of cluster dose, enabling the prediction of cell survival across different radiation types without explicitly introducing RBE. In this sense, cluster dose offers a more physically grounded and unified framework for describing radiation effectiveness. However, we acknowledge that alternative mathematical models might better describe the relationship between cluster dose and survival for certain circumstances, as is the case for absorbed dose. This is an area that warrants further investigation with larger datasets.

Cluster dose is uniquely amenable to ID-based RTP, compared to alternative nanodosimetric quantities, especially when cluster dose is closely associated with cell survival. Since cluster dose is analogous to absorbed dose (clusters per unit mass as opposed to energy deposited per unit mass), clinicians may easily adapt to reviewing cluster dose distributions. Since cluster dose and absorbed dose are both proportional to charged particle fluence, established methods for optimization of absorbed dose distributions may be applied to optimization of cluster dose distributions, as previously described. In addition, cluster dose constraints may be adopted from established absorbed dose constraints. Although cluster dose may be used alone in the plan optimization, ID-based planning may be more acceptable to clinicians if cluster dose were combined with other quantities such as voxel-averaged *I*_p_ and absorbed dose approaches (e.g. RBE-weighted dose) in plan optimization (see, e.g. equation (23) in Faddegon *et al* 2023).

A comprehensive set of measurements showing the dependence of cell survival on cluster dose was used to quantitatively evaluate preferred *I*_p_ for application in ID-based RTP. These datasets cover a wide range of particles (from protons to argon ions), particle energies, and doses, for two different human cell lines. The availability of detailed information on beam delivery and irradiation conditions enabled precise reproduction of the experimental setups for accurate *I*p and cluster dose calculations. Additionally, the datasets were derived from experiments conducted by independent researchers at two different institutions, which reduced the possibility of coincidental agreement on the preferred *I*_p_. However, although results are compelling, one must not draw rigorous conclusions on the selection of preferred *I*_p_ from these results alone, as discussed shortly.

Three different statistical approaches were used for quasi-continuous evaluation in the dispersion of these cluster dose survival curves. The result was quantitative agreement in the determination that cluster dose survival curves based on the preferred *I*_p_ in aerobic condition, *C*_5_, and in hypoxic conditions, *C*_7_, exhibited the least dispersion. These findings augment the results of our previous study that found preferred *I*_p_ by evaluating their association with cell survival using plots of survival against *I*_p_ at constant fluence (Faddegon *et al* 2023). In that work, as seen in figure 6, we found that larger cluster sizes were associated with cell survival in hypoxic cells than in aerobic cells, explained by the lower probability of an ionization to be converted to a DNA strand break and induce cell death in hypoxic conditions (Schulte *et al* 2003). In this work, we found that for the preferred *I*_p_ from our previous work, approximately the same survival is expected for the same cluster dose, independent of particle type and energy. This may allow for the prescription of the same cluster dose regardless of whether the radiation beam consists of a mixture of different particles and energies, as is typically the scenario in particle therapy. Unlike RBE-based models, which require specific weighting factors and often lead to varying predictions of isoeffective dose with no established consensus, cluster dose does not require for a RBE-like weighting factor. When using the preferred *I*_p_ values, the resulting cluster dose survival curves exhibit the lowest variability in survival for a given cluster dose. By evaluating the difference in chi-squared values between individual (per particle) and combined (all particles) LQ fits (equation (13)), we demonstrate that a preferred *I*_p_ exists for which the dependence of the cluster dose survival relationship on radiation quality is minimized. In other words, the relationship between cluster dose and survival can be described by a single LQ fit that is independent of particle type and energy. This implies that, while in RBE-weighted dose approaches the alpha and beta coefficients of the LQ model depend on both cell type and radiation type, in the cluster dose framework these coefficients become cell-specific constants, with reference coefficients applicable across all radiation qualities. The present work illustrates the potential of using cluster dose survival curves to identify a preferred Ip definition compatible with this formulation. Overall, this work provides further evidence for use of the ID approach in ion RTP, by converting the spatial pattern of ionization at the nanoscale, characterized by the *I*_p_, to cluster dose.

**Figure 7.**
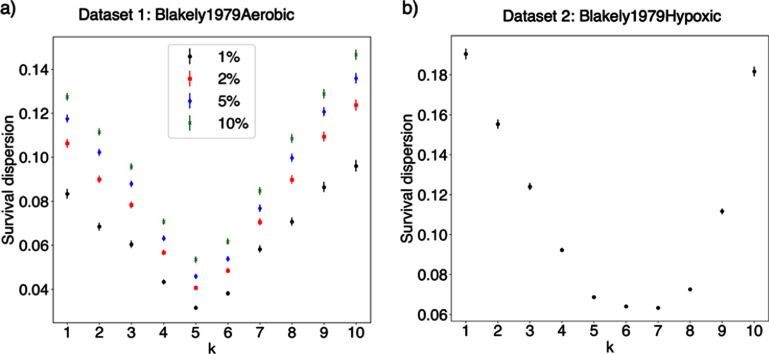
Dispersion of survival data for various *C_k_* (with *k* from 1 to 10) computed with Method 1 and different window sizes. (a) Window sizes (i.e. number of points included in the sliding window) are defined as the percentage (1%, 2%, 5%, and 10%) of points in the entire dataset. (b) Windows sizes are defined as the adjacent points within 5% of the cluster dose at each datapoint. Datasets 1 and 2 are shown as examples. The error bars correspond to one standard deviation.

The statistical methods proposed for this evaluation are not only useful for the study of cluster dose survival curves, but also for identification of preferred *I*_p_ from plots of survival and other biological endpoints against *I*_p_ at constant fluence. Method 1 (the moving window method) may be used for any biological endpoint, independent of the trend, since it does not require fitting a function to the data. In this work, we set the window size as function of the size of the dataset. We showed results for a window size of 1% of the total number of points in the dataset or 3 datapoints, whichever encompasses more points. Window sizes of 2%, 5%, and 10% of the size of the dataset also resulted in a minima for the preferred *I*_p_ (figure 7). Thus, the method was insensitive to window size.

We also investigated the use of a variable window for Method 1. Each window, centered on each datapoint, included the adjacent datapoints within 5% of the cluster dose. The rationale behind the use of this approach was to quantify the variation in survival considering the uniformity constraints of a given quantity (e.g. cluster dose), typically used in RTP. This approach also led to the selection of the same *I*_p_ (see figure 7(b)). However, the use of a variable window is limited to datasets in which the density of data points along the abscissa (cluster dose axis) is high and nearly constant and presented here as an example of application.

Contrary to Method 1, Method 2 (the calculation of residuals from a fit) and Method 3 (BIC from a fit) are of interest for studying endpoints that are expected to follow a known response curve. Then, Methods 2 and 3 allow us to consider the fit of the data to that response model in the evaluation of preferred *I*_p_. The residuals from Method 2 are useful to compute the dispersion of the data to a model that is well known (e.g. the LQ model for survival data in this work). Conversely, the BIC may be appropriate when comparing different models for a given biological endpoint whose trend is not well characterized. The BIC method considers the model that best fits the data and its simplicity, i.e. it includes a penalty term for the number of model parameters to avoid overfitting with very complex models. In the present article, both approaches (Method 2 and Method 3) were used as proof-of-principle examples. In the case of using cell survival as the biological endpoint, they are statistically correlated since both rely on the residuals from the same model (an LQ fit). Both methods are presented to provide investigators with a choice that best aligns with the biological endpoint under investigation. For evaluating the association between cluster dose and cell survival, which, based on the results of this study, follows a LQ (or almost linear) dependence, Method 2 (residuals from a generic LQ fit) may be the most appropriate. This method incorporates a global fit across radiation qualities using common alpha and beta coefficients, making it potentially useful for survival prediction in preclinical and future clinical contexts. This may also be useful for future radiobiological modeling efforts, since this approach facilitates comparisons with current RBE-weighted or absorbed dose frameworks, facilitating a transition to ID-based planning methodologies.

Note that the proposed approach for selecting the preferred *I*_p_ definition is compatible with any dataset and potential future revisions of the fICSD database. The fICSD data used in this study was originally developed for demonstration purposes (Faddegon *et al* 2023). The methodology underlying the generation of the ICSD database, including the influence of source and scoring volume geometry, is the focus of a separate technical study currently under review. While geometric factors can influence cluster dose distributions, an in-depth methodological evaluation is beyond the scope of the present work. The theoretical framework presented here is general and can be applied independently of the specific method used to compute the underlying database, whether using the current or future revised geometries and radiation transport configurations. To address potential concerns regarding border effects in the current geometry, we performed additional simulations using periodic boundary conditions for charged secondary particles. These simulations confirmed that applying periodic boundary conditions had no significant impact on the computed average fICSD compared to the baseline configuration, supporting that border effects are negligible for the present work. Further, our geometric conditions at the nanometer level are quite realistic, reproducing a relatively large segment of a chromatin fiber filled with randomly oriented DNA segments of biologically relevant size (10 base pairs).

The approach established in this work is intended for use in future studies involving larger and more diverse datasets, such as those in the publicly available PIDE database (Friedrich *et al* 2013). While *C_k_* was selected here because previous work demonstrated its strong association with survival, the methodology is general and can be applied to any *I*_p_ formulation, including more complex definitions such as linear combinations of different *C_k_* values, as previously proposed (Conte *et al* 2023). The present *I*_p_ selection, based on a limited dataset, demonstrates the use of cluster dose survival curves to quantitatively discriminate between different *I*_p_ definitions, which represents the scope and novelty of this paper.

The clinical translation of cluster dose would be facilitated should there be a means to measure *I*_p_ and cluster dose, first to benchmark Monte Carlo simulation, additionally to provide quality assurance and calibration in clinical settings. Currently, experimental nanodosimetry is primarily limited to gas-based detectors (Rucinski *et al* 2021). Such detectors can provide experimental benchmarks of fICSD in gas, but currently fICSD simulations are mostly limited to liquid water. For clinical settings, advancements in solid-state detectors, such as CMOS-based technologies, may enable the development of millimeter-scale detectors for nanodosimetric applications in the future (Lee *et al* 2020). While direct measurement of cluster dose in clinical settings remains unfeasible, fluence, the other component of cluster dose, can be experimentally verified. This is analogous to RBE-weighted dose, where the absorbed dose component can be measured in clinical settings, while the biological component (RBE) relies on predictive models.

## 5. Conclusion

Overall, this work advances the process of integration of nanodosimetric quantities in ion RTP. Cluster dose survival curves were shown to have considerable value in selecting ionization parameters (*I*_p_) for their use in RTP optimization. Results of analysis of a limited set of published cell survival data with rigorous statistical approaches found that *I*_p_ exhibiting the highest degree of association with cell survival for different particles having the same fluence resulted in the least dispersion in cluster dose survival curves. This least dispersion methodology is suitable to quantitatively identify *I*_p_ with this important trait for ID-based RTP for published and new radiation effect measurements.

## Data Availability

All data that support the findings of this study are included within the article (and any supplementary information files).
